# Patterning of High-Viscosity Silver Paste by an Electrohydrodynamic-Jet Printer for Use in TFT Applications

**DOI:** 10.1038/s41598-019-45504-5

**Published:** 2019-06-24

**Authors:** Thi Thu Thuy Can, Tuan Canh Nguyen, Woon-Seop Choi

**Affiliations:** Department of Display Engineering, Hoseo University, Asan, Chungnam 31499 Korea

**Keywords:** Electronic properties and materials, Electronic devices

## Abstract

Electrohydrodynamic (EHD) jet printing has a variety of benefits compared to conventional inkjet techniques, such as high resolution and the ability to work with high-viscosity pastes. In this work, Ag nanoparticles with 4000 cPs were chosen because they are printable on various substrates for electronic devices. The effects of additive on the high-viscosity Ag paste formulation were investigated, and pattern lines narrower than 100 *μ*m were achieved by EHD-jet printing with an average sheet resistance of 0.027 Ω □^−1^. Furthermore, solution-processed oxide TFTs were fabricated with EHD jet-printed Ag electrodes for the first time. The electrical properties obtained were a current ratio of 1.5 × 10^6^, a mobility of approximately 1 cm^2^ V^−1^ s^−1^, a threshold voltage of 21.5 V, and a subthreshold slope of 3.05 V dec^−1^.

## Introduction

Direct-write techniques have been investigated extensively for making fine patterns in micro- or nano-scale. This technique is low cost, high speed, contact-free operation and very environmentally friendly manufacturing processes compared to conventional methods^1,2^. Among the large number of direct-write techniques, the inkjet method is widely known for fabricating printed electronic devices. The benefits of inkjet printing are on-demand jetting and high throughput with a high jetting frequency of 10–100 kHz. However, because of the large size of droplets or patterns, it is not easy to meet the demanding requirements in case of high-density and conductive printed circuit boards or thin-film transistors (TFTs)^3,4^.

For printed electronics, silver nanoparticles attracted a great interest for the many applications because of their thermally and electrically conductive, solvent resistant, and adhesive to most materials. Many studies have concentrated on the direct printing of silver nanoparticles with very low viscosity for the fabrication of electrically functional microstructures^5–7^.

Electrohydrodynamic (EHD) jet printing is a technique with very potentials for directly patterning functional materials onto many substrates. Its ability to make patterns and use in thin film deposition can support the fabrication of the electronic devices through a single technique^8^. EHD jet printing has received attention as a non-contact printing technique with a unique ability to produce fine patterns with either a large-diameter nozzle or ultra-fine nozzle^9–11^.

Some of our previous studies on EHD jet printing demonstrated high-resolution patterns compared to spin-coating and ink-jet techniques. The TFT characteristics attained using an EHD jet printer were much better than those obtained with spin-coating technology. An EHD jet-sprayed TFT showed a threshold voltage of 7.17 V and a very high on-to-off ratio of 10^7^ ^12^. Moreover, the electrical properties of the pattern printed from the EHD jet printer surpassed those of the conventional ink jet technique. An EHD jet-printed ZTO TFT showed a field-effect mobility of 9.82 cm^2^ V^−1^ s^−1^, on-to-off current ratio of 3.7 × 10^6^, and threshold voltage of 2.36 V at 500 °C^13^.

Just few researchers have examined multi-nozzle EHD jet printing techniques to overcome the difficulties of the inkjet technique^14,15^. A three-nozzle-EHD inkjet printing head was used to fabricate conductive lines of silver colloidal ink on glass substrate simultaneously. However, the process was quite complex, and average printed track widths of 140 ± 5 μm were obtained^16^.

In this study, silver line patterns from high-viscosity Ag paste formulations were produced using an EHD jet printer and applied in electronic devices. Sharp and continuous lines with a width of 100 μm or less were obtained for the first time using a very inexpensive steel needle that required no treatments. ZTO TFTs with EHD-fabricated Ag source and drain electrodes were also successfully manufactured with acceptable electrical characteristics.

## Results and Discussion

### Effect of tip height on patterns

**Figure 1:**
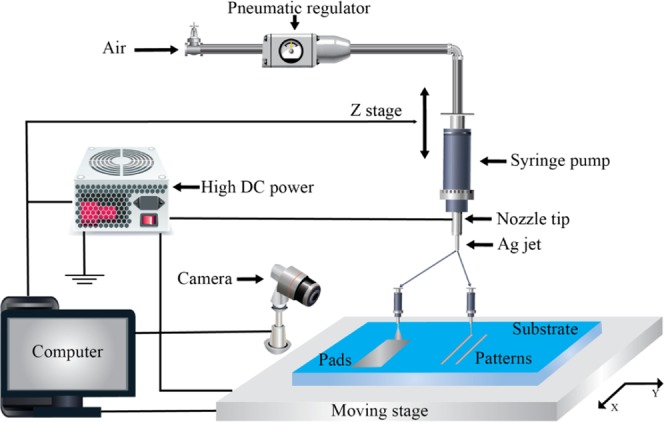
Configuration of experimental setup used for EHD jet printing.

**Figure 2:**
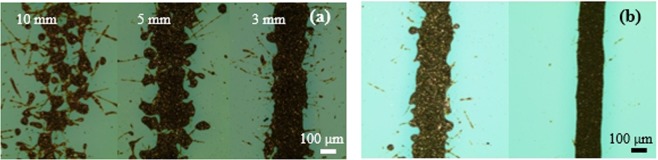
(**a**) Optical microscopy images of EHD-jetted Ag patterns with various distances between the substrate and nozzle tip of 10, 5 and 3 mm. (**b**) Optical microscopy images of EHD-jetted Ag patterns: without PGMEA (left) and with PGMEA (right) (volume ratio of Ag nano-particles: silveray: PGMEA = 100:1:1).

The first main factor that affects the quality of a pattern line is the distance h between the nozzle tip and the substrate surface, which is called the tip height in Fig. 1. To investigate the dependence between h and the silver line characteristics, Ag paste was jetted using three tip heights of approximately 3, 5, and 10 mm. At 10 mm, no pattern in the line shape can be obtained because the distance is large, as shown in Fig. 2a. The multi-jet mode and/or ramified-jet mode occurred, and there was no obvious change in the shape of the patterns. The patterns did not seem to be continuous, and there were some small holes in the lines. With a tip height of 3 mm, the lines covered the substrate well, but there was still splashing on two sides of the patterns. When moving the tip closer to ITO surface, it is not safe for the tip and the substrate because of the increased electrostatic force between the nozzle and conductive stage. Stabilizing and optimizing tip height is critical for microscale applications in EHD jet printing. Thus, the tip height should be around 3 mm to obtain acceptable patterns.

### Effect of additive on patterns

In general, commercially nanoparticle precursor conductive inks with low viscosity are produced with low evaporation rate binder solvents in order to get rid of the blockage in the nozzle tips in printing technology. As a result, it requires long drying times for printed patterns which limits to mass production. In this work, silver paste with relatively high viscosity of 4,000 cPs can easily be accumulated at the narrow print-head. Therefore, finding an additive to prevent material obstruction and allow for promoting evaporation rate as well is a key component in the process of preparing the electrically conductive ink. Many ingredients can be added to improve the quality of silver paste quality, such as a weak solvent, binder, stabilizer, and so on. We found that the desired neat patterns could not be obtained with only the original silver nanoparticle pastes (the left image of Fig. 2b). To optimize the conductive paste for inkjet printing, several groups have used solvents with a high vapor pressure to allow for quick drying time, such as hexane and toluene^17–19^. Among many solvents used in commercial conductive inks, propylene glycol monomethyl ether acetate (PGMEA) is known as a solvent commonly used in inks, coating, and cleaner in the semiconductor industry, primarily for the application of surface adherents. It is featured by high vapor pressure of 2.8 mmHg at 20 °C, boiling point of 146 °C and low viscosity of 1.1 mPas. A fast drying-conductive ink was developed for inkjet printing conductive tracks, and the solvent played a main role because of the relatively high vapor pressure compared to those of solvents used in commercial conductive inks^20^.

Therefore, PGMEA was added to the silver nanoparticle paste in this research to obtain neat and smooth edges. A tiny amount of PGMEA enormously enhanced the silver patterns, as shown in the right image of Fig. 2b. Particularly, smaller width and good coverage of the substrate were achieved due to the adhesion between the paste and ITO glass.

### Effect of stage speeds and voltages on patterns

**Figure 3:**
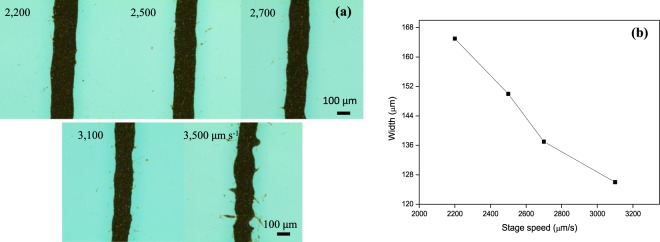
(**a**) Optical microscopy images of EHD-jetted Ag patterns with various stage speeds from 2,200 to 3,500 μm s^−1^. (**b**) Pattern width was measured from 4 lines corresponding to different stage speeds with a ratio of original silver paste:silveray:PGMEA mixture of 100:1:1.

The same conditions used to obtain neat silver wires were applied but with a faster stage speed ranging from 2000 to 3500 μm s^−1^. The volume ratio of silver paste: silveray (solvent): PGMEA (additive) in the silver mixture was 100:1:1. The width of the printed silver decreased when the stage velocity increased, as illustrated in Fig. 3a. The reason is that the stage moved quickly and the resulted in much less time for silver paste approach surface. The maximum speed should be lower than 3500 μm s^−1^ to obtain straight and sharp lines, as shown in Fig. 3b. The smallest width achieved was 126 μm.

**Figure 4:**
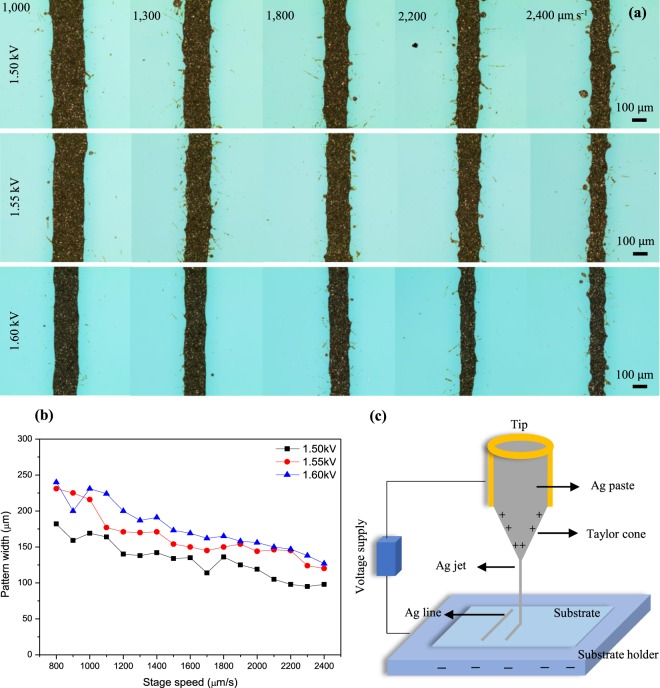
(**a**) Optical microscopy images of EHD-jetted Ag patterns prepared with original silver paste:silveray:PGMEA mixture of 100:1:1.5. Pattern lines were printed at different stage speeds from 1,000 to 2,400 μm s^−1^ with applied voltages of 1.50, 1.55, and 1.60 kV. (**b**) Pattern width as a function of applied voltage as substrate speed was increased from 800 to 2400 μm s^−1^. (**c**) Schematic diagram Taylor cone-jetting mode.

Figure 4a illustrates the dependence of the pattern width on the voltage and stage speed. The experiment was carried out with a material volume ratio of 100:1:1.5 (original silver paste:silverary:PGMEA), and the range of pressure was 80–90 kPa. With a satisfactory applied voltage, the paste from the nozzle tip forms a Taylor cone, and a fluid jet is emitted from the metallic tip (Fig. 4c). When no electric field (0 kV) was applied to the tip, silver paste accumulated at the end of the tip due to repulsive pressure from the syringe. Moreover, a paste droplet formed and stayed right at the tip mouth in a hemispherical shape due to surface tension. Increasing the applied voltage gave rise to a conical shape because the surface tension is sufficiently higher than the repulsive electrostatic force. Right after obtaining a Taylor cone, the smallest and sharpest patterns were achieved with a width of around 100 μm at a voltage of 1.50 kV in the range of speed of 2100 to 2400 μm s^−1^. In general, as the applied voltage changes from 1.50 kV to 1.60 kV, the pattern becomes larger (Fig. 4b). The highest widths were close to 230 μm and were seen when jetting the silver paste at 800–900 μm s^−1^ for two cases of 1.55 and 1.60 kV. It seemed that there was no much difference in the line width because of the small range of applied voltage (1.50, 1.55, and 1.60 kV). The silver lines became narrower when decreasing the applied voltage. The desired patterns had width of 100–150 μm at voltages of 1.50 to 1.60 kV in the speed range of 2100 to 2400 μm s^−1^. When raising the voltage to more than 1.85 kV, the electric force started standing out from the surface tension, and the droplet quickly changed into a spray shape, and it was not sufficient to draw the pattern lines.

### Effect of pressures on patterns

Air pressure is one of the most important factors when jetting high-viscosity ink like silver paste. To obtain the cone-jet mode, there must be appreciable pressure. Fig. S1 in the Supplementary Information (S.I.) shows two critical notes. First, higher pressure means there is stronger force to push the paste through the nozzle tip, which corresponds to a lower speed range compared to the lower-pressure case. The combination of high pressure and high stage speed makes it easy for silver paste to splash on the substrate. Second, just enough pressure is needed to obtain a narrow width. In the case of silver paste with a volume ratio of 100:1:1(original silver paste:silveray:PGMEA), a tiny pattern width of 125 μm could be achieved with 80-kPa-of-pressure.

### Effect of material ratios on patterns

After many experiments, the optimal process parameters and suitable ratio of materials were tested to obtain pattern with a width of 100 μm with a pressure around 80 kPa and a voltage of 1.50 to 1.65 kV. With a volume ratio of 100:1:1.5 (original silver paste: silveray: PGMEA), the best results were achieved with continuous, sharp, and even patterns. As shown in the microscope images in Fig. S2 in the S.I. we obtained narrow patterns of approximate 90 μm with just a small amount of PGMEA added to form the Ag paste.

### Nanoview images

**Figure 5:**
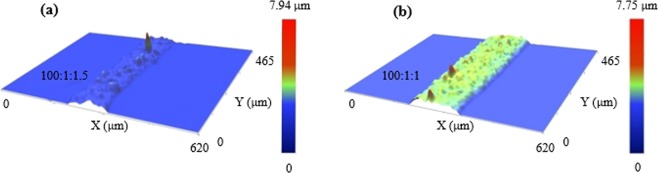
Nano-view image of patterns printed from different silver paste formulations (**a**) 100:1:1.5 and (**b**) 100:1:1 at the same other conditions: v_stage_ = 2,500 μm s^−1^, U = 1.8 kV, h = 3 mm, P = 80 kPa.

Nanoview equipment has a user-friendly design and is easy to use with good resolution and high-accuracy measurements. This kind of system was used to obtain more information about the topology of patterns with 3D morphologies and clearly show the roughness, flatness, thickness, and line profiles. The silver patterns were measured using the jetting conditions corresponding to the two previous cases of ratios. The pattern width measured using the Nano-view system was similar to those in the microcopy image. The thickness of the pattern with ≈1.5% PGMEA is lower than that with ≈1% PGMEA (Fig. 5). This was suitable according to theoretical knowledge regarding the function of PGMEA. The peak-to-valley distance of the pattern with 1.5% PGMEA and the one with 1% PGMEA were 1.38 and 2.05 μm, respectively. However, the root mean square (RMS) roughness was just slightly different of 0.37 (100:1:1.5) and 0.40 μm (100:1:1), respectively, as shown in Table 1.

**Table 1:** Morphological information of printed Ag patterns from different formulation pastes.

Materials Volume Ratio	Thickness (μm)	Width (μm)		RMS Roughness (μm)	Peak to Valley (μm)
		Nanoview	Microscope		
100:1:1.5	0.8	105.0	104.0	0.37	1.38
100:1:1.0	1.4	157.7	147.0	0.40	2.05

Beside surface features and dimensional information given above, cross-sectional view of line patterns was depicted. When an ink drop or line dries on a solid surface, it leaves a ring-like deposit along the edge, known as the coffee-ring effect. This phenomena is due to the enhanced solvent evaporation near the edges compared to that at the center as the substrate is heated. As a result, solute particles move toward the pattern edges to replace fluid lost and accumulate there. That behavior is commonly seen in inkjet printing technique because of easy evaporated solvent making up a large portion in the ink. Basically, the coffee-ring effect leads to non-uniform morphology, and then affects performance of printed devices. Therefore, it is necessary to improve the uniformity of printed patterns by negligible coffee-ring effect. In this study, silver pattern lines fabricated using EHD jet printing technique revealed the reverse coffee-ring effect. Instead of typical concave shape in coffee-ring behavior, the cross-sectional area indicated a convex shape of the printed lines. This inverse is determined by the amount of additive and the viscosity of paste. A tiny amount (1% or 1.5% volume ratio) of PGMEA additive formulated into Ag paste with relative high paste viscosity can lead Ag nanoparticle’s motion slow, and then prevent the formation of coffee-ring.

### Electrical properties of Ag patterns

In order to analyze the electrical properties of the EHD jet-printed silver pattern, Ag paste was printed in a 5-mm square shape on the top of ZTO/SiO_2_/Si because the narrow pattern lines were not suitable for measurement by four-point probe equipment. The formation of silver paste was slightly different from the narrow printed patterns on ITO glass in the previous case because of the different surface properties between ITO and ZTO film. Silver paste (60:1:1 of Ag nano-particles:silveray:PGMEA) was EHD-sprayed in cone-jet mode with a tip height of 12 mm, pressure of 250 kPa, and voltage of 3.8 kV, as shown in Fig. S3 in the S.I. At high tip-to-substrate gap and high electric forces, the mist of small Ag droplets existed from stable jet due to the electrostatic repulsive forces. This phenomenon is called electrospray atomization which was utilized for the fabrication of Ag area in this study. This was followed by drying at 150 °C on a hot plate. The optical image taken at the boundary between Ag film and ZTO layer as indicated in Fig. S3b in the S. I. The sheet resistance of printed Ag area was around of 0.027 Ω □^−1^. In comparison to results from the inkjet printing technique, this value is 4 times lower than that from our previous work on screen-printed Ag and 10 times less than that of an Ag area obtained using inkjet printing and post-treatment with infrared sintering^21,22^. This means that the conductivity was improved in the present research. The results demonstrate the high potential of EHD jet-printed electronics. The results also show that the sheet resistance depends on the duration of sintering on the hot plate and several processing parameters.

### TFT characteristics

**Figure 6:**
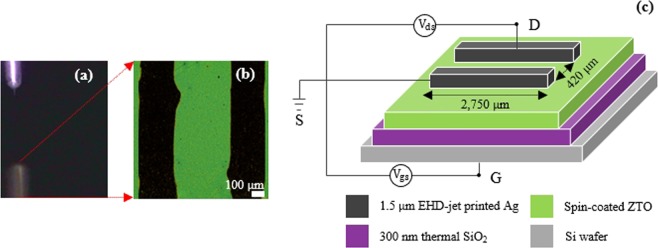
(**a**) Camera image of cone-jet mode for printing Ag electrodes. (**b**) Microscope image of the top view and (**c**) schematic representative of the cross-sectional view of a ZTO TFT with printed Ag S/D.

**Figure 7:**
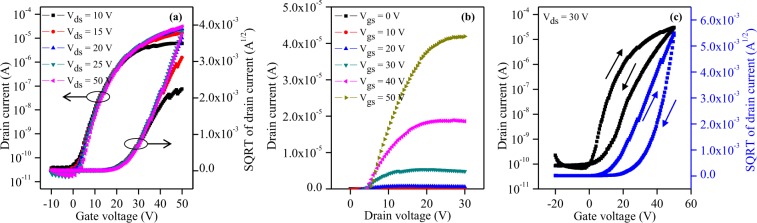
(**a**) Transfer characteristics at V_ds_ from 10 to 50 V. (**b**) Output characteristics of ZTO TFT with EHD jet-printed Ag source and drain electrodes. (**c**) Hysteresis characteristics evaluated based on sweeping the gate bias from −20 to 50 V and back with drain voltage of 30 V.

A ZTO-based TFT was designed with a channel length of 420 μm and channel width of 2,750 μm, as schematically indicated in optical top view (Fig. 6b) and cross-sectional view image (Fig. 6c). The Ag S/D electrodes, around 1.5-μm-thickness (from 100:1:1 paste formation as mentioned in Fig. 5b) and 220-μm-line width, were directed designed through EHD jet printing via cone-jet mode (Fig. 6a). Herein, the line width of Ag electrodes and the channel length were designed to create favorable conditions for measuring TFT characteristics and to prevent the bulging at two ends of printed lines. The transfer properties of the TFT was measured while sweeping the gate bias from −10 to 50 V. Figure 7a shows the transfer curves of the device for different drain voltages. The electrical performance of the ZTO TFTs can be deduced from the original data shown in transfer curves, including the threshold voltage (V_th_), subthreshold slope (SS), and saturation mobility (μ_sat_). The threshold voltage and mobility were calculated from the slope of $\sqrt{{{\rm{I}}}_{{\rm{ds}}}}$ versus V_gs_ according to the TFT model in the saturation regime:

$${{\rm{\mu }}}_{{\rm{sat}}}=\frac{2{\rm{L}}}{{\rm{W}}.{{\rm{C}}}_{{\rm{i}}}}{(\frac{\partial \sqrt{{{\rm{I}}}_{{\rm{ds}}}}}{\partial {{\rm{V}}}_{{\rm{gs}}}})}^{2}$$

C_i_ is the capacitance per unit area of 300-nm-SiO_2_ (gate insulator, 10^−8^ F cm^−2^), and L and W are the length and width of the channel, respectively.

The characteristic parameters showed a mobility of 0.97 cm^2^ V^−1^ s^−1^, threshold voltage of 21.5 V, subthreshold slope of 3.05 V dec^−1^. and current ratio (I_on_/I_off_) of 1.5 × 10^6^ (V_ds_ ≥ 2.0  V), which are comparable to those of conventional solution-processed ZTO TFTs obtained with other methods for the deposition of the source and drain^21,23^. While this mobility can be compared to that of our previous research on spin coated-(1.05 cm^2^ V^−1^ s^−1^) and inkjet processed-(1.2 cm^2^ V^−1^ s^−1^) ZTO TFT with Ag screen-printed S/D^21^, it was lower than that of other solution processed ZTO with vacuum deposited S/D^24^. The improved mobility value of their TFT devices may be explained due to two main reasons: (1) tighter contact between ZTO and metal S/D, and better connection of metal particles in vacuum and (2) lower work function (ϕ)^24^ difference between S/D electrode and ZTO semiconductor compared to our devices, in which electron carriers have to overcome to be injected into the semiconductor layer (ϕ_A1_ = 4.28 eV^25^, ϕ_ZTO_ = 4.35 eV^23^, ϕ_Ag_ = 4.6 eV^25^). Turn-on voltage, which is gate voltage required to turn on/off the transistor in a switching application, is of around 4 V with our device. Moreover, as the drain-source voltage increased to around 20 V, the threshold voltage shifted to the right from 21.5 to 25 V. Instability in response to drain bias might be attributed to some reasons: (1) electron trapping at the interface between the ZTO layer and SiO_2_ gate insulator, (2) electron injection into SiO_2_ layer and (3) creation of acceptor-like defect states. This revealed that the operation of the ZTO-based TFTs with EHD jet-printed contacts remained in enhancement mode with the drain voltage. The OFF currents were identical for all conditions of source-drain voltage (around 4 × 10^−11^ A). On the other hand, the ON current obtained at V_ds_ = 10 V was obviously lower than those at higher drain voltages. The increase in the ON current at higher V_ds_ could be explained by the higher number of free carriers in the active layer resulting in easier aggregation of the charges at the semiconductor/dielectric interface. With a drain voltage of 15 V, the ON state levels were almost the same (over 10^−5^ A), regardless of the drain bias, although a small positive shift could be seen. Similarly, a subthreshold slope (SS) and a saturation mobility were unchanged with higher drain bias, which might indicate that few defect states were generated at the interface of the active layer/gate insulator. These acceptable mobility (1–10 cm^2^ V^−1^ s^−1^) and large on/off current ratio (>10^5^) are sufficient to meet the brightness and resolution requirements for backplanes in display applications.

The output characteristics of the ZTO TFTs with printed silver source and drain electrodes were measured while sweeping V_ds_ from 0 to 30 V for various V_gs_ at intervals of 10 V. Figure 7b exhibits clear current saturation and a pinch-off region. The drain current increases rapidly with increasing drain-source voltage at a positive gate bias which demonstrates that electrons are mainly generated, especially V_gs_ > 30 V. However, a crowding effect also occurred at low drain voltages resulting from the contact resistance, which should be sufficiently low for source-to-drain conduction. This behavior needs to be minimized to improve the contact by applying a new formulation, process enhancement, or other techniques in future device designs.

Obvious hysteresis could be seen in the transfer curves when the gate bias was swept from −20 and 50 V and back at various source-to-drain voltages of 15 to 30 V, as shown in Fig. 7c for the case of 30 V of V_ds_. An applied drain voltage of 20 V or higher clearly leads to reduced width of the hysteresis. There positive shifts in the threshold voltage of 27.4, 17.3, and 16.5 V corresponding V_ds_ of 15, 20, and 30 V, respectively. The hysteresis phenomena in the transfer behavior might be from three mechanisms: charge injection at the ZTO active layer/SiO_2_ dielectric interlayer, charge trapping at the SiO_2_/Si gate interface, and the redistribution of charged defects in the Si layer.

This hysteresis is attributed to the uncertainty of the source and drain contacts as a result of some accumulation of Ag nanoparticles during process. Because the injected charges are responsible for the hysteresis of the transfer property in the device, a hysteresis-free transfer curve should be obtained by allowing them to disappear before measuring the source and drain current. In further applications, with this type of hysteresis phenomenon, it is essential to minimize the interface trap density or to design a pixel circuit that is insensitive to the hysteresis feature for highly effective TFT-based panels, such as those based on active-matrix organic light-emitting diodes (AMOLEDs).

## Conclusion

The high viscosity of silver paste may be a problem with a majority of jet printing techniques. By adding an effective additive in the Ag paste formulation, the deposition of high viscosity silver nanoparticles on ITO glass was successfully achieved by the EHD jet printing method. To obtain less than 100 μm width, the experimental conditions were determined as a 3-mm spacing between the substrate and the electrode nozzle, applied voltage range of 1.5 to 1.6 kV, stage speed of 1200 to 2500 μm s^−1^, and the the pressure of 80–90 kPa.

The silver patterns fabricated were well organized and had electrical properties with an average sheet resistance of approximately 0.027 Ω $\square$^−1^. Solution processed ZTO-based TFTs were also achieved with EHD-jet printed Ag source and drain, which had a mobility of 0.97 cm^2^ V^−1^ s^−1^, and on-off current ratio of over 10^6^. The method for obtaining the narrow patterns could be used in the fabrication of microelectronics.

## Methods

Silver nanoparticle paste (AD-V7-108) with a high viscosity of 4000 cPs was obtained from the Exax Inc. The silver paste formulation was prepared for EHD jet printing by mixing silver nanoparticles with a corresponding solvent (silveray) and propylene glycol methyl ether acetate (PGMEA) as a useful additive. Immediately afterwards, the mixture was evened out by stirring for 15 minutes at room temperature. To evaluate EHD-jet printability of silver paste and investigate effect of parameters on Ag patterns, Ag lines were printed on ITO glass first. Before the EHD jet printing, ITO coated glass substrates were cleaned using deionized (DI) water, acetone, and isopropyl alcohol, followed by UV/O_3_ treatment to remove any organic residuals and improve the wettability.

For making high viscous Ag patterns, an EHD jet printer with a precision three-axis motorized stage, a pneumatic dispensing system with a precision pressure regulator, and a high-voltage was used. To reduce the vibration noise, the xyz moving stage was placed on a table that is completely isolated from vibration. In this configuration, three stabilized stages configured in the x, y, and z directions were used to move the main stage (in the x and y directions) and nozzles (in the z direction). The pattern lines were deposited on a 10-cm square glass substrate coated with a 500 μm-thick-layer of ITO. The ground plate was mounted on the xy motorized stage to control the substrate speed during processing. The range of the stage speed was 800 to 8000 μm s^−1^. Because of high-viscosity of the Ag nano-particle paste, a pneumatic equipment was added to the system to provide pressure for the EHD jet printing process and to keep the required flow speed. A digital high-speed microscope video camera was used to monitor the printing process. DinoCapture software and a micrometer were installed to observe and accurately control the stage speed and substrate position. A steel tip was employed on the plastic nozzle with 100-μm inner diameter and 230-μm outer diameter. The steel tip has relatively low cost compared to brittle glass capillary nozzles.

The bias flowing between tip and substrate were manually varied from 1.5 to 2.0 kV using a high-voltage power supply to obtain cone-type jetting. Different distances from the nozzle head to the glass substrate were tested (3, 5, and 10 mm) for the deposition of the silver paste. After patterning the lines, the samples were annealed at a temperature of 150 °C for 10 minutes in air using a conventional hot plate. To estimate the electrical property of printed silver, the square Ag film with the size of 5 × 5 mm^2^ was spray printed on the top of ZTO/SiO_2_/Si.

A solution-processed ZTO TFT device was fabricated with a common architecture of a staggered bottom-gate and top-contact (BGTC) to investigate the use of the EHD jet-printed silver patterns as electrode parts. The precursors for the active layer were zinc acetate dihydrate, tin (II) chloride, and acetylacetone as a stabilizer (mole ratio of 1:1:1), which were dissolved in 2-methoxyethanol to form a 0.3 M sol-gel solution with stirring for 6 h at room temperature. The semiconductor layer was spin-coated on a p-doped Si wafer with 300-nm thick thermally grown SiO_2_ substrate. The substrate was treated with UV/O_3_ for 30 min before doing the experiment to increase the hydrophilic ability. After covering the SiO_2_ gate insulator with the ZTO precursor solution, the film was dried by pre-baking at 150 °C for 10 min to evaporate, and followed by annealing at 550 °C for 1 h in a box furnace to decompose the zinc acetate and tin chloride, as well as to form a metal oxide layer. Silver source and drain electrodes were EHD jet-printed with a mixture of Ag nanoparticles, silveray, PGMEA as a volume ratio of 100:1:1.

Optical Microscopy (Olympus-BX51M) was utilized to analyze 2D image of the printed Ag. 3D image and thickness of the patterns was determined using a Nano-View NV-2000 measurement system (Nano-system Company). To determine the sheet resistance of the printed silver, a silver area was EHD jet-printed and then measured using a four-point probe resistivity measurement system (Jandel Miniature Cartridge probe on AIT equipment). All electrical and stability characteristics of the TFTs were measured in a dark box at room temperature using a semiconductor parameter analyzer (Keithley 4200).

## Supplementary information

Supplementary Information
