# Supplementary material for: Patterning of High-Viscosity Silver Paste by an Electrohydrodynamic-Jet Printer for Use in TFT Applications
Source: Sci Rep. 2019 Jun 24;9:9180. doi: 10.1038/s41598-019-45504-5 (PMC6591279; doi:10.1038/s41598-019-45504-5)
Supplement: Supplementary file 1 — Supplementary Information [file 41598_2019_45504_MOESM1_ESM.docx]

***Supplementary Information***

**Patterning of High-Viscosity Silver Paste by an Electrohydrodynamic-Jet Printer for Use in TFT Applications**

*Thi Thu Thuy Can, Tuan Canh Nguyen, and Woon-Seop Choi^*^*

Department of Display Engineering, Hoseo University, Asan city, Chungnam 31499, Korea

**Corresponding Author**

* Email: [wschoi@hoseo.edu](mailto:wschoi@hoseo.edu)

Fig. S1 Optical images of Ag lines as different pressures are applied.

Fig. S2 Optical images of Ag lines from different material volume ratios.

Fig. S3 Schematic of experiment set up for printing Ag area of 5$\times$5 mm^2^ at 1.2 cm tip height and 3.8 kV applied voltage.


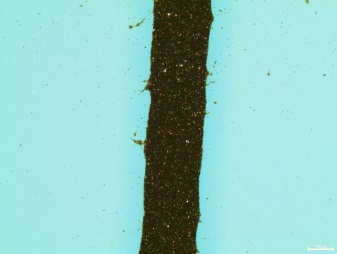

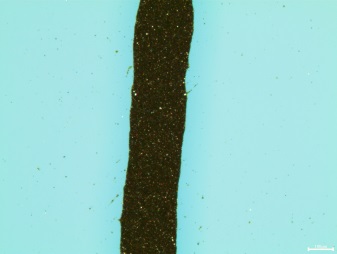

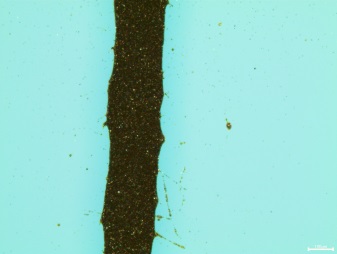


100$\mu$m


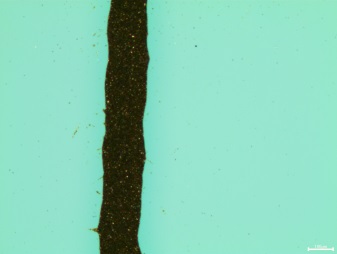

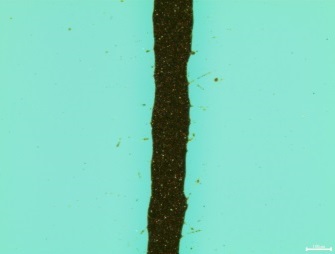


100$\mu$m


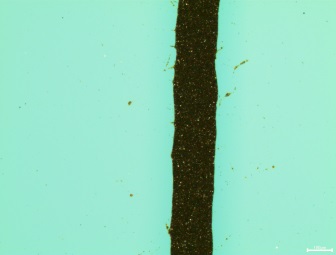


**(b)**

**Fig. S1** (**a**) Optical images of jet-printed Ag patterns from Ag nanoparticles:silveray:PGMEA mixture of 100:1:1 when paste was pushed out at pressure of 95 kPa with stage speed from 1,700 to 1,900$\mu$m s^-1^ (top) and 80-kPa case with stage speed from 2,300 to 3,100$\mu$m s^-1^ (bottom). (**b**) Dependence on stage speeds of pattern width with different pressures.

1,700

1,800

1,900

2,300

2,400

3,100

$\mu$m s^-1^

**(a)**

95 kPa

80 kPa


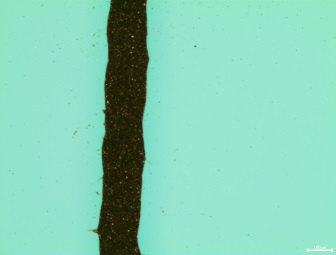


100$\mu$m


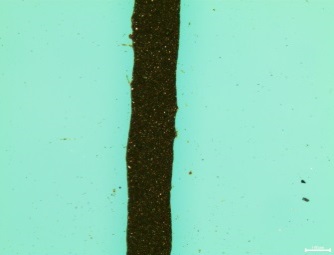

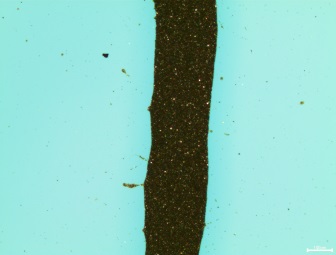

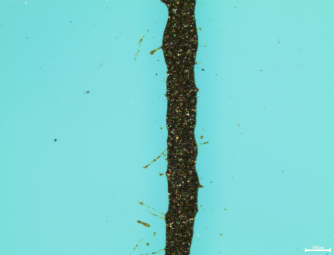


100$\mu$m


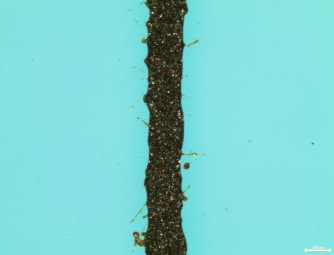

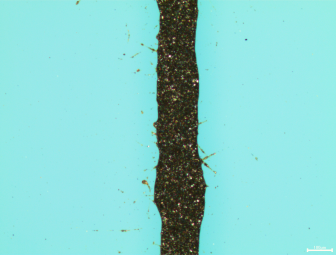


**(b)**

**Fig. S2** (**a**) Top optical images of jet printed Ag patterns from Ag nanoparticles:silveray:PGMEA mixture of 100:1:1 and bottom images for 100:1:1.5 with stage speed from 1,600 to 2,400 $\mu$m s^-1^. (**b**) Dependence on stage speeds of pattern width with different material volume ratios.

1,600

2,000

2,400

$\mu$m s^-1^

100:1:1

100:1:1.5

**(a)**


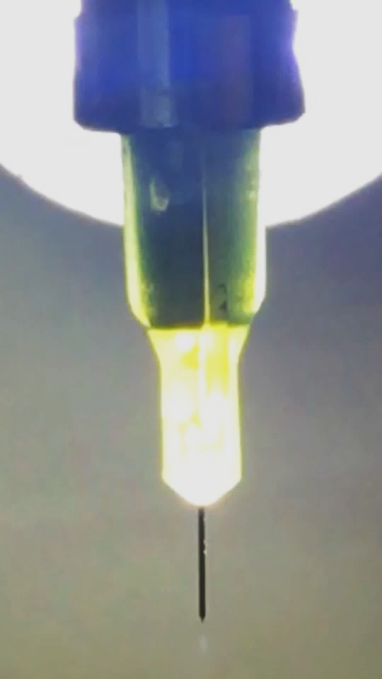

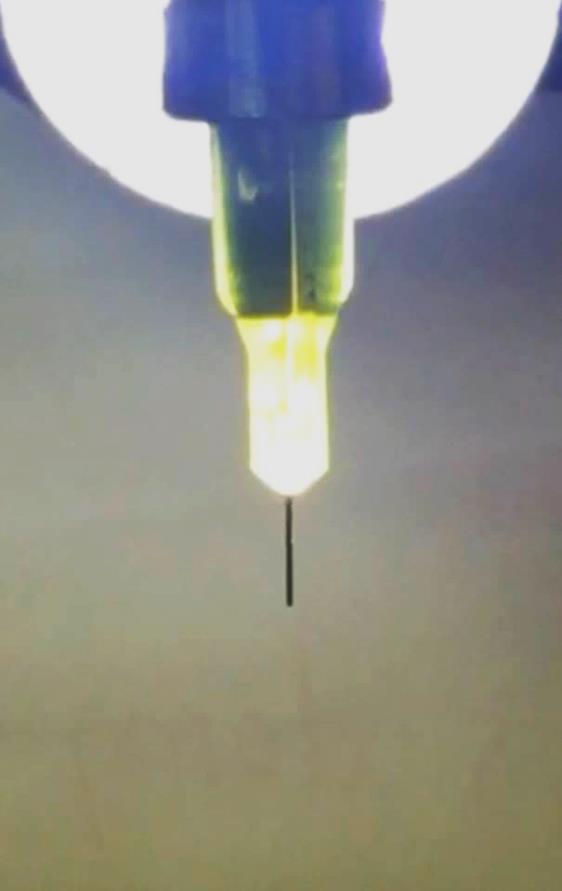


0 V

3.8 V

1.2 cm


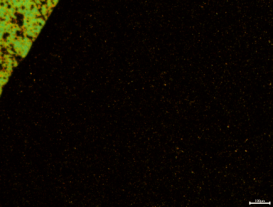


100 $\mu$m

**Fig. S3** (**a**) Schematic of experiment set up for printing Ag area of 5$\times$5 mm^2^ at 1.2 cm tip height and 3.8 kV applied voltage. (**b**) Microscope image of printed Ag pattern area on ZTO layer.

5 mm

5 mm

**(b)**

**(a)**

**Ag**

**ZTO**
